# A single cytosine deletion in the *OsPLS1* gene encoding vacuolar-type H^+^-ATPase subunit A1 leads to premature leaf senescence and seed dormancy in rice

**DOI:** 10.1093/jxb/erw109

**Published:** 2016-03-19

**Authors:** Xi Yang, Pan Gong, Kunyu Li, Fudeng Huang, Fangmin Cheng, Gang Pan

**Affiliations:** ^1^Department of Agronomy, Zijingang Campus, Zhejiang University, Hangzhou 310058, PR China; ^2^Institute of Crop and Nuclear Technology Utilization, Zhejiang Academy of Agricultural Sciences, Hangzhou 310021, PR China

**Keywords:** *Oryza sativa* L., *OsPLS1*, premature leaf senescence, ROS, SA, seed dormancy, vacuolar-type H^+^-ATPase.

## Abstract

Leaf senescence is a programmed developmental process orchestrated by many factors, but its molecular regulation is not yet fully understood. In this study, a novel *Oryza sativa* premature leaf senescence mutant (*ospls1*) was examined. Despite normal development in early seedlings, the *ospls1* mutant leaves displayed lesion-mimics and early senescence, and a high transpiration rate after tillering. The mutant also showed seed dormancy attributable to physical (defect of micropyle structure) and physiological (abscisic acid sensitivity) factors. Using a map-based cloning approach, we determined that a cytosine deletion in the *OsPLS1* gene encoding vacuolar H^+^-ATPase subunit A1 (VHA-A1) underlies the phenotypic abnormalities in the *ospls1* mutant. The *OsPSL1/VHA-A1* transcript levels progressively declined with the age-dependent leaf senescence in both the *ospls1* mutant and its wild type. The significant decrease in both *OsPSL1/VHA-A1* gene expression and VHA enzyme activity in the *ospls1* mutant strongly suggests a negative regulatory role for the normal *OsPLS1/VHA-A1* gene in the onset of rice leaf senescence. The *ospls1* mutant featured higher salicylic acid (SA) levels and reactive oxygen species (ROS) accumulation, and activation of signal transduction by up-regulation of *WRKY* genes in leaves. Consistent with this, the *ospls1* mutant exhibited hypersensitivity to exogenous SA and/or H_2_O_2_. Collectively, these results indicated that the *OsPSL1/VAH-A1* mutation played a causal role in premature leaf senescence through a combination of ROS and SA signals. To conclude, *OsPLS1* is implicated in leaf senescence and seed dormancy in rice.

## Highlight

The *OsPLS1* locus that encodes VHA-A1 in rice, was identified by map-based cloning. *OsPLS1/VHA-A1* is involved in premature leaf senescence and seed dormancy.

## Introduction

Vacuolar H^+^-ATPases (VHAs) are highly conserved enzyme complexes making up 6.5–35% of the total tonoplast protein mass in different plants. These enzymes are distributed in vacuoles and other membrane-bound organelles such as the Golgi apparatus and endoplasmic reticulum (Schumacher and Krebs, 2010). There are two distinct VHA subcomplexes, namely V0 and V1. The cytosolic V1 subcomplex, which consists of eight subunits (A–H), is responsible for ATP hydrolysis, while the transmembrane V0 subcomplex, with six subunits (a, c, c', c'', d, and e), is responsible for proton translocation (Schumacher and Krebs, 2010). It is conceivable that VHAs are involved in the generation of a proton gradient and membrane potential which are required to energize secondary transport across the tonoplast (Marty, 1999). In plants, VHAs have proved to be important in several cellular processes and physiological responses, including male gametophyte development (Dettmer *et al.*, 2005), nutrient storage (Krebs *et al.*, 2010), environmental stress tolerance (Wang *et al.*, 2011), glucose signaling (Cho *et al.*, 2006), plant growth (Zhou *et al.*, 2016), and seed germination (Cooley *et al.*, 1999). In addition, several subunits of the VHA complex are implicated in cell growth and death in humans and in plant species (Zhan *et al.*, 2003; Krebs *et al.*, 2010; Tang *et al.*, 2012). Consistently, inhibition of VHA has been reported to induce cell death in human cell cultures such as HeLa cells (Zhan *et al.*, 2003) and hypoxic tumor cells (Graham *et al.*, 2014). It is not surprising that a *vha-a2 vha-a3 Arabidopsis* double mutant, which lost 85% VHA activity compared with its wild-type plants, features stunted growth, lower fertility, and development of necrotic lesions at the leaf tips and flowers (Krebs *et al.*, 2010; Tang *et al.*, 2012).

Vacuolar H^+^-ATPase subunit A (VHA-A) is a catalytic component of VHA subcomplex V1. In most plants, VHA-A is encoded by two different genes, *VHA-A1* and *VHA-A2* (Schumacher and Krebs, 2010). In tomato, despite the 96% amino acid sequence identity of their products, *VHA-A1* is ubiquitously expressed in various tissues and organs, while *VHA-A2* expression is mainly restricted to the roots and fruits (Bageshwar *et al.*, 2005). Antisense-mediated inhibition of *VHA-A2* gene expression was reported to retard seed formation in tomato (Amemiya *et al.*, 2006). In sugar beet, *VHA-A* gene expression declines in aging leaves (Lehr *et al.*, 1999). In *Arabidopsis*, deficiency in *VHA-A* leads to complete male and partial female gametophytic lethality (Dettmer *et al.*, 2005). Recently, Zhang *et al.* (2013) reported that RNAi-mediated inhibition of *OsVHA-A* resulted in an increase in stomatal aperture and density, and higher susceptibility to drought and salt stress in transgenic rice. Overall, these studies support a crucial role for *VHA-A* in plant cell growth and death, and seed development. In plants, cell death caused by senescence of leaves has been thought to be a type of programmed cell death (PCD) (Zhou and Gan, 2009). Moreover, two distinctive subdomains of apoptotic-like PCD in different placentochalazal layers were observed in maize seed coat development (Kladnik *et al.*, 2004). However, until now, little has been elucidated on the involvement of *VHA-A* in leaf senescence and seed dormancy.

Reactive oxygen species (ROS) and salicylic acid (SA) have long been considered important signaling molecules and key regulators of plant PCD during defense response against abiotic and biotic stress (Herrera-Vásquez *et al.*, 2015). Moreover, it was demonstrated that ROS signals, in particular H_2_O_2_ and O_2_^–^, are involved in upstream and/or downstream SA signaling in response to stress (Mori *et al.*, 2001; Herrera-Vásquez *et al.*, 2015). SA is a type of phenolic phytohomone known to have a variety of functions in plant cell growth, stomatal aperture, respiration, seed germination, and seedling development (Hayat *et al.*, 2012). SA is derived from chorismate via two distinct pathways, the isochorismate synthase (ICS) pathway and the phenylalanine ammonia-lyase (PAL) pathway. Once synthesized, SA usually undergoes a number of modifications including glucosylation, methylation, and amino acid conjugation (Dempsey *et al.*, 2011). Of note, SA conjugation with glucose at the hydroxyl group probably forms SA 2-*O*-β-d-glucoside in the cytoplasm (Dean *et al.*, 2005). SA glucoside can be transported from the cytoplasm into the vacuoles in soybean and tobacco cells where it may serve as inactive storage for later release of free SA (Dean *et al.*, 2005). Interestingly, inhibition of the VHA activity was shown to decrease the SA 2-*O*-β-d-glucoside uptake into vacuoles in soybean cells (Dean and Mills, 2004). Moreover, both SA and H_2_O_2_ signaling are known to induce the expression of the *WRKY* transcription factors genes *WRKY53*, -*54*, *-70*, and -*72* (Zhang and Zhou, 2013; Zhou *et al.*, 2013; Bakshi and Oelmüller, 2014), which were presumed to be implicated in the regulation of leaf senescence. Furthermore, exogenous SA (Zhao *et al.*, 2012) or H_2_O_2_ (Li *et al.*, 2015) could accelerate senescence of detached leaves. However, so far, our understanding of the precise contribution of ROS and/or SA signal to leaf senescence is relatively poor. In particular, the *VHA-A*-dependent leaf senescence in relation to ROS and SA signaling remains to be further investigated.

In this study, a novel premature senescence rice mutant, termed *Oryza* s*ativa premature leaf senescence 1* (*ospls1*), was isolated through γ-ray radiation-mediated mutagenesis. The *ospls1* mutant leaves manifested lesion-mimics and premature leaf senescence after tillering. The putative *OsPLS1* gene was identified through a map-based cloning strategy. *OsPLS1* encodes the vacuolar H^+^-ATPase A1-subunit (OsPLS1/VHA-A1). We found that *OsPLS1/VHA-A1* mutation resulted in leaf senescence through ROS and SA signaling, and seed dormancy due to shallow and compact micropyles in the glumellae and to abscisic acid (ABA) signaling. Our experimental data showed that *OsPLS1/VHA-A1* is responsible for leaf senescence and seed dormancy in rice.

## Materials and methods

### Plant materials and growth conditions

The *ospls1* mutant was obtained from the ^60^Co γ-irradiated *indica* restore line N142. The original control N142, M_8_ generation seeds of *ospls1*, and the *japonica* cultivar 02428 were grown in the paddy field. For map-based cloning, *ospls1* and 02428 were used to produce an F_2_ population.

### Measurement of photosynthesis

The rate of photosynthesis, stomatal conductance, and transpiration rate were measured on intact single-flag leaflets of *ospls1* and its wild type using a LI-6400 portable photosynthesis system (LI-COR, Lincoln, NE, USA) (Pan *et al.*, 2012).

### Measurement of chlorophyll content, and endogenous SA and ABA levels

Chlorophyll was extracted from the flag leaves in 10ml of 80% acetone for 16h in the dark and was determined by measuring the absorbance at 652nm (Arnon, 1949).

Endogenous SA were extracted from the flag leaves at the preliminary heading stage and the fully expanded leaves of the seedlings. Endogenous ABA was extracted from the germinated seeds. SA and ABA levels were quantified using a HPLC-ESI-MS/MS system (Pan *et al.*, 2010).

### ROS accumulation and ROS-scavenging enzyme assays

3,3'-Diaminobenzidine (DAB) and nitroblue tetrazolium (NBT) staining for determination of ROS accumulation were performed on the flag leaves at the preliminary heading stage (Qiao *et al.*, 2010). H_2_O_2_ contents in the seedling leaves and the flag leaves were measured using an H_2_O_2_ Assay Kit (Nanjing Jiancheng Bioengineering Research Institute). The O_2_^−^ level was measured by monitoring nitrite formation from hydroxylamine according to Wang and Luo (1990). The activities of superoxide dismutase (SOD) and catalase (CAT) were determined according to previously described methods (Zhou *et al.*, 2013).

### Exogenous SA and/or H_2_O_2_ treatment

The *ospls1* mutant and its wild-type seeds were sterilized and germinated on 1/2 MS medium (Murashige and Skoog, 1962) with or without SA or H_2_O_2_ at 27 °C with a 16h light/8h dark cycle. The shoot and root lengths were measured after 6 d of growth. In a separate experiment, detached flag leaves were cut into ~4cm segments and immersed in water with or without 5mM SA and/or 100mM H_2_O_2_. The samples were incubated at 25 °C in darkness for 2.5 d, and then photographed.

To check the *OsPLS1* expression during SA treatment, the wild-type N142 were germinated and grown in Yoshida solution (Yoshida *et al.*, 1976) in the greenhouse at 28 °C/24 °C day/night with a 16h light/8h dark cycle. Seedlings at the three- to four-leaf stage were incubated in water or 5mM SA. Five plants were collected at each time point at 0, 0.75, 1.5, 3, 6, 12, and 24h after treatment for RNA isolation.

### Seed germination

The intact grains and dehulled grains were either germinated under complete submergence in water with or without oxygen supplementation provided via an air pump, or on pre-wetted filter papers, or they were germinated in water solution with ABA, GA_3_, or fluridone. Assays were performed in a growth chamber with a 16h light/8h dark cycle at 27 °C. Germination is defined as a radicle length >1mm . Germination counts were made twice a day for 7 d. The results presented are the means of the germination percentages obtained after various time periods in three replicates.

### Microscopic observations of the leaf and dry mature seeds

For scanning electronic microscopy (SEM) observation, 2mm^2^ leaf tissues were taken from the middle part of the flag leaves and samples were prepared (Zhang *et al.*, 2013). For examining micropyles of the seeds, mature dry grains were directly sputter-coated with platinum. Leaf samples and seeds were observed and photographed by SEM (Hitachi TM-1000).

For transmission electron microscopy (TEM) observation, flag leaves were fixed with 2.5% glutaraldehyde. Ultrathin samples were made and viewed and photographed by TEM (Hitachi H-7650) (Zhou *et al.*, 2013).

### Genetic analysis and molecular mapping

For genetic analysis, the leaf phenotypes of F_1_ and F_2_ plants were observed from crossing *ospls1* to N142 and 02428, respectively. The F_2_ population derived from the cross between 02428 and *ospls1* was used for bulk segregant analysis (BSA), and preliminary and fine mapping of the *OsPLS1* locus. Using 10 mutant plants obtained in the F_2_ population, BSA was first performed for preliminary genetic mapping using 10 simple sequence repeat (SSR) markers from the http://www.gramene.org/ website and insertion/deletion (InDel) markers (Shen *et al.*, 2004) belonging to each of the 12 rice chromosomes. After BSA, additional molecular markers surrounding the preliminary location were used to screen recombination events from 315 F_2_ individuals for fine-mapping. To fine-map the *OsPLS1* gene, five markers (S1–S5) (Supplementary Table S1 at *JXB* online) were developed for fine-mapping based on DNA sequence differences between *indica* and *japonica* rice varieties.

### Sequence analysis of the candidate genes

Based on the physical map of the *OsPLS1* gene, ORFs defined by the two markers S3 and S5 were used to analyze their functions in RAGP (http://rapdb.dna.affrc.go.jp/). The full-length genomic DNA sequence of each candidate gene was amplified from N142 and *ospls1* using PrimerSTAR polymerase (Takara Bio Inc.). PCR products were sequenced using an ABI Prism Model 3700 sequencer in Sunny Co. Ltd., Shanghai, China. Sequence alignment was performed to identify the sites of mutation with DNAMAN sequence analysis software.

### dCAPS marker analysis

To confirm the single nucleotide repeat (SNP) in the *ospls1* mutant, derived cleaved amplified polymorphic sequence (dCAPS) analysis was performed using the dCAPS primers dCAPS-OsPLS1 (Supplementary Table S2). The 1bp mismatched primer was designed using the mutation point of the *OsPLS1* allele through the web server program dCAPS Finder 2.0 (http://helix.wustl.edu/dcaps/dcaps.html). Amplified products were digested with *Apa*I that recognized the SNP.

### Enzyme activity measurements

Leaf tonoplast membrane proteins were extracted from the flag leaves (Krebs *et al.*, 2010). V-ATPase activity of 10 μg of microsomal membranes was determined as phosphate release (Krebs *et al.*, 2010) after 40min incubation at 28 °C. A 10 μg aliquot of bovine serum albumin was used as the negative control, and the reactions were terminated by adding 40mM citric acid. The VHA activity was assayed according to previously described methods (Zhang *et al.*, 2013).

### Cloning construct and rice transformation

The NOS terminator from the vector pBI121 (Clontech) was inserted into the multiple cloning site of the binary vector pCAMBIA1300 after double digestion with *Sac*I and *Eco*RI to form the new vector pC1300-Nos. Then the full-length cDNA of *OsPLS1* and the promoter region ranging from 2252bp upstream of the translation initiation codon of *OsPLS1* was amplified with specific primers (Supplementary Table S2). Using the In-Fusion HD cloning Kit (Clontech), the full-length cDNA and its promoter were cloned into pC1300-Nos which was digested in advance with *Pst*I. The clones were further verified by sequencing; the resulting clone was named pOsPLS1 and introduced into *Agrobacterium* strain EHA105 for transformation of the *ospls1* mutant (Pan *et al.*, 2006). The regenerated plants were confirmed by PCR analysis using the *OsPLS1*-specific primers and the hygromycin resistance-specific primers (Supplementary Table S2).

### RNA isolation and real-time reverse transcription–PCR (RT–PCR) analysis

Total RNA was extracted from the seedlings, roots, flowers, shoots, and flag leaves with Trizol reagent (Invitrogen). First, total RNA was treated with DNase I for removal of the possible contamination of genomic DNAs. Then, first-strand cDNAs were synthesized with 2 µg of total RNA in a 20 µl volume using oligo(dT_23_VN) and HiScript^®^ II reverse transcriptase (Vazyme biotech, USA). For real-time PCR analysis, the 20 µl aliquots of cDNAs were diluted to 200 µl, 2 µl of which was added to 12.5 µl of SYBR Premix Ex Taq II (Takara, 2x) and 0.4 µM of each primer in a final 25 µl reaction. PCRs were performed on the Roche LightCycler 480. The qRT-PCR conditions were 95 °C for 30s, followed by 40 cycles of 95 °C for 5s, 60 °C for 30s, and 72 °C for 30s. *UBQ*5 was used as the endogenous control gene. The relative expression levels were calculated using the 2^–∆∆CT^ method (Schmittgen and Livak, 2008). The primers used are listed in Supplementary Table S3.

## Results

### Lesion-mimics in the leaf and premature leaf senescence phenotype of the *ospls1* mutants

**Fig. 1.**
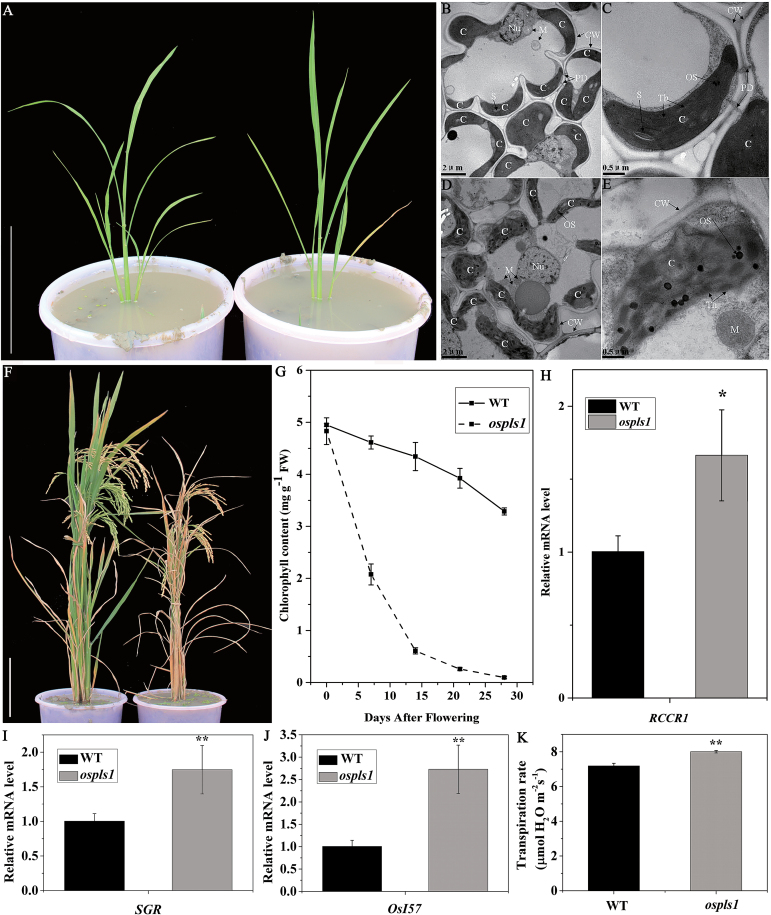
The *ospls1* mutant phenotypes. (A) Plants at the tillering stage. Scale bar=20cm. (B–E) Chloroplast ultrastructure of the wild type (B, C) and *ospls1* (D, E). C, chloroplast; CW, cell wall; M, mitochondrion; N, nucleus; OS, osmiophilic body; S, starch grain; Th, thylakoid; PD, plasmodesma. (F) Plants on day 28 after flowering. Scale bar=20cm. (G) The total chlorophyll content of the flag leaf after flowering. (H–J) Expression of *RCCR1*, *SGR*, and *OsI57*. (K) Transpiration rate of the flag leaf at the preliminary heading stage. Values are means ±SD of four biological replicates. *P<0.05, **P<0.01 (*t*-test)

Using γ-ray radiation mutagenesis, we obtained the *ospls1* mutant. Compared with its wild type, the *ospls1* mutant did not display noticeable phenotypic abnormalities including leaf appearance at the early developmental stage (prior to the four- to five-leaf seedlings). However, the *ospls1* mutant exhibited lesion-mimics and early senescence after tillering, initially from the tips of the lower leaves, followed by exacerbated red-brown lesions rapidly spreading downward to cover the whole leaf blade except the upper 1–2 fully expanded leaves and heart leaves (Fig. 1A). At the jointing and early heading stage, all of the leaves except the flag leaves of the *ospls1* mutant manifested early senescence (Supplementary Fig. S1); and in the flag leaves of the *ospls1* mutant at the early heading stage, TEM indicated that grana began to break down and osmiophilic plastoglobules increased in size and number (Fig. 1B–E). Relative to the wild type, the *ospls1* mutant leaves were significantly withered at both the flowering and grain-filling stages (Fig. 1F), which led to reduction of several agronomic traits, especially the panicle number, plant height, panicle length, and 1000-grain weight (Supplementary Table S4). During the post-flowering stage, the total chlorophyll levels in both the wild-type and *ospls1* mutant flag leaves became progressively lower, with that in the latter decreasing even more rapidly (Fig. 1G). As a result, the chlorophyll content of the mutant was only 2.9% of that in the wild type on day 28 after flowering (Fig. 1G).

Considering rapid chlorophyll loss in the *ospls1* mutant during senescence, we analyzed the messenger abundance of two chlorophyll degradation-related genes, red chlorophyll catabolite reductase 1 (*RCCR1*) and stay-green (*SGR*), and one senescence-related gene *OsI57*, a positive marker for leaf senescence. As shown in Fig. 1H–J, a significant difference in the transcriptional levels of *RCCR1*, *SGR*, and *OsI57* expressed in the flag leaves was observed between the *ospls1* mutant and its wild type, with strikingly elevated expression in the *ospls1* mutant.

**Fig. 2.**
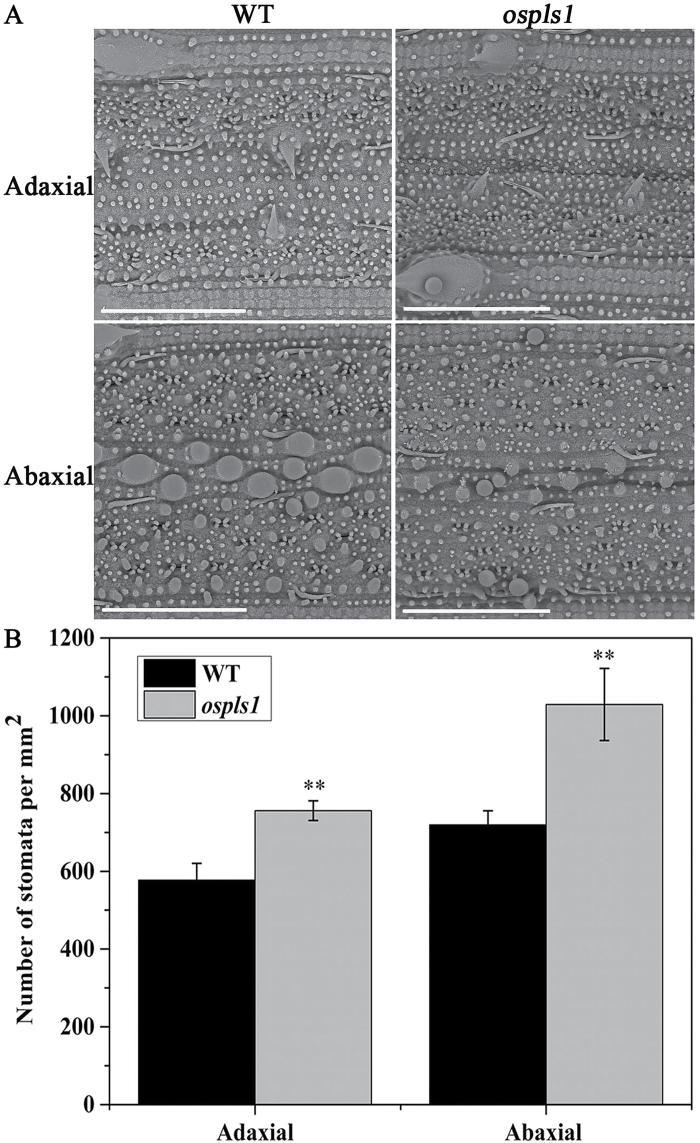
SEM analyses of the flag leaf. (A, B) SEM observation (A) and stomatal density (B) of the abaxial and adaxial surface. Scale bar=60 μm. Error bars indicate the SD (*n*=20). **P<0.01 (*t*-test).

Premature leaf senescence would lead to a reduction of the photosynthesis rate, a lower grain filling rate, and yield loss in crops (Wu *et al.*, 2012). At the preliminary heading stage, despite a comparable net photosynthesis rate and stomatal conductance between the *ospls1* mutant and its wild type (Supplementary Table S5 at *JXB* online), the transpiration rate of the flag leaves was 11.43% higher in the *ospls1* mutant (Fig. 1K). SEM observation further revealed that the stomatal densities in the flag leaves of the *ospls1* mutant increased by 42.98% and 30.95% on the adaxial and abaxial surfaces, respectively, compared with its wild type (Fig. 2A, B). Due to high stomatal densities, the *ospls1* mutant leaves lost more water than those of the wild type during dehydration (Supplementary Fig. S2).

### Genetic analysis and fine-mapping of *OsPLS1*

For genetic analysis, two F_2_ populations were developed from crossing *ospls1* to its wild-type N142 and 02428, respectively. All F_1_ plants showed the wild-type phenotype. In both of the F_2_ populations, the wild-type and mutant phenotypes segregated at a typical 3:1 ratio (Supplementary Table S6). Together, these results suggested that the phenotype of *ospls1* was controlled by a single recessive nuclear gene.

**Fig. 3.**
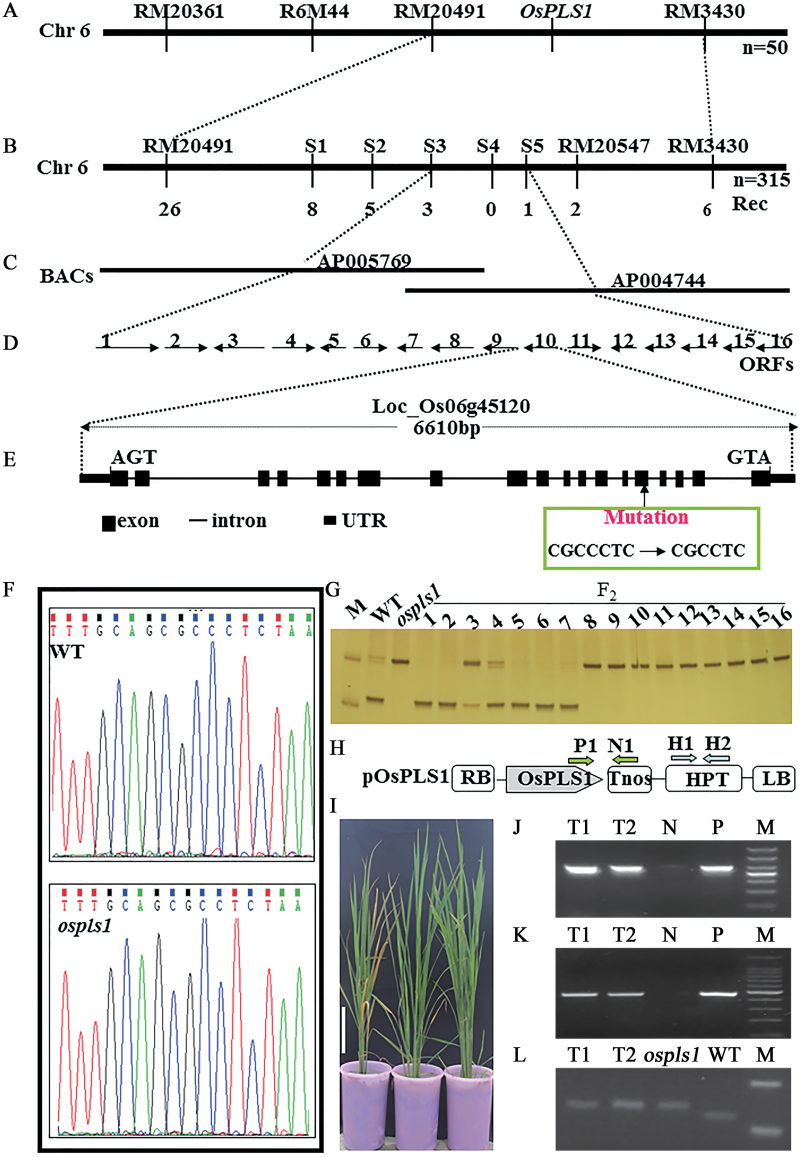
Map-based cloning of the *OsPLS1* locus. (A, B) Preliminary mapping (A) and fine-mapping (B) of the *OsPLS1* locus with SSR and InDel markers. (C) BAC clones located at the fine-mapping region. (D) Candidate genes in the 85kb region identified by fine-mapping. (E) *OsPLS1* gene structure at the genomic level. Nineteen exons and 18 introns are indicated by black rectangles and lines, respectively; a single cytosine deletion was identified in the fifth exon. (F) Sequence confirmation of the mutation site in the *ospls1* mutant. (G) dCAPS detection of the *ospls1* mutation sites. M, 20bp DNA ladder. 1–7 and 8–16 represent the F_2_ individuals with the wild-type and mutant phenotype, respectively. (H) Schematic diagram of the pOsPLS1 construct for genetic complementation in the *ospls1* mutant. LB, left border; RB, right border; *HPT*, hygromycin phosphotransferase gene; Tnos, the nopaline synthase gene. (I) Phenotype of the transgenic rice at the tillering stage. (J, K) PCR detection of the *HPT* gene (J) and exogenous transgene (K). P, plasmid of pOsPLS1; N, non-transgenic *ospls1* mutant; T1–T2, transgenic events; M, marker. (L) dCAPS detection of the endogenous *ospls1* mutant allele. T1–T2, transgenic events; *ospls1*, non-transgenic *ospls1* mutant; WT, wild-type plants. M, marker.

Next, the F_2_ population derived from *ospls1*/02428 was employed for mapping of the *OsPLS1* locus. Among the 550 molecular markers, 178 showed polymorphism between *ospls1* and 02428, and were further applied for analysis of the linkage relationship with the *OsPLS1* locus. Further, BSA revealed that three SSR makers (RM20361, RM20491, and RM3430), and one InDel marker (R6M44) located on the long arm of chromosome 6 displayed segregation distortion with an early-senescence phenotype (Fig. 3A), pointing to preliminary localization of the *OsPLS1* locus on chromosome 6. Linkage analysis of 315 F_2_ recessive early-senescence individuals derived from *ospls1*/02428 confirmed that the *OsPLS1* locus was located between RM20491 and RM3430 (Fig. 3A).

For fine mapping of the *OsPLS1* locus, a high-resolution physical map was constructed through the analysis of five new markers in 32 recombinants derived from RM20491 and RM3430. With the physical map, the *OsPLS1* locus was eventually limited to an 85kb region between two new markers S3 and S5 (Fig. 3B) in the BAC (bacterial artificial chromosome) clones AP005769 and AP004744 (Fig. 3C).

### *OsPLS1* encodes a vacuolar-type H^+^-ATPase subunit A1

The 85kb interval bordering markers S3 and S5 contained 16 putative genes in the Rice Annotation Project Database (http://rapdb.dna.affrc.go.jp/) (Fig. 3D), including one (LOC_Os06g45120) encoding the vacuolar H^+^-ATPase A-subunit (VHA-A) and another (LOC_Os06g45110) encoding a DNA-binding protein. Of the 16 putative genes, only these two genes were potentially relevant to observations on the *ospls1* mutant: the single-stranded DNA-binding protein WHIRYl was reported to repress *WRKY53* expression and delay leaf senescence in Arabidopsis (Miao *et al.*, 2013), while VHAs were shown to be involved in cell death (Tang *et al.*, 2012). Thus, these two genes were considered as the primary candidates for the *OsPLS1* mutation. Sequence analysis showed that the candidate LOC_Os06g45120 differed between the *ospls1* mutant and its wild type. The LOC_Os06g45120 gene contains 19 exons and 18 introns, and encodes a protein with 620 amino acids (Fig. 3E). The sequencing result showed that a C was deleted at the 312^th^ nucleotide in the coding frame of the *ospls1* mutant, leading to a frameshift and premature stop of translation in the fifth exon (Fig. 3E, F). To verify this mutation, we further employed a dCAPS analysis to rule out sequencing errors. The genomic fragment spanning mutated sites using the dCAPS marker CAPS-PLS1 (Supplementary Table S2) was amplified and digested by *Apa*I. The results further proved that this deletion of C at the 312^th^ nucleotide existed in the *ospls1* mutant (Fig. 3G). Furthermore, the co-segregation of the polymorphism with the recessive mutant phenotype was also observed when the recessive F_2_ individuals and the parents were compared in the simultaneous dCAPS analysis (Fig. 3G). These results suggested that the *VHA-A* gene might be the *OsPLS1* locus. Since rice VHA-A is encoded by two different genes (Schumacher and Krebs, 2010), LOC_Os06g45120 and LOC_Os02g07870, we rename LOC_Os06g45120 as *OsPLS1/VHA-A1* and LOC_Os02g07870 as *VHA-A2*.

### Complementation test of *OsPLS1/VHA-A1*

To obtain more convincing evidence that *OsPLS1/VHA-A1* corresponds to the *OsPLS1* locus, a genetic complementation test was conducted. pOsPLS1 containing *OsPLS1/VHA-A1* full-length cDNA and its own promoter was introduced into the *ospls1* mutant through *Agrobacterium*-mediated transformation (Fig. 3H). PCR analysis of all eight plants obtained from six independent transgenic events confirmed the presence of both a hygromycin resistance gene and the exogenous *OsPLS1* gene in the recipient genome (Fig. 3J, K), while the endogenous mutant allele in the pOsPLS1 transgenic lines was confirmed by CAPS-PLS1 (Fig. 3L). Moreover, when the positive transgenic lines were grown in the field, the lesion-mimics and premature leaf senescence phenotype seen in the *ospls1* mutant were completely rescued in the six independent pOsPLS1 transgenic lines (Fig. 3I). Therefore, we concluded that LOC_Os06g45120 is the *OsPLS1/VHA-A1* gene.

### Expression analysis of *OsPLS1/VHA-A1*

**Fig. 4.**
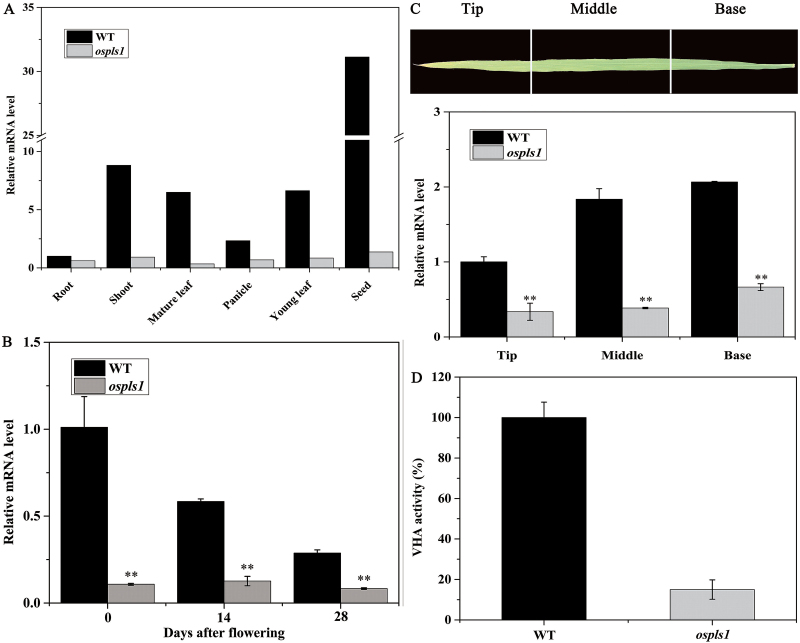
Analysis of *OsPLS1/VHA-A1* gene expression and VHA activity in *ospls1*. (A) *OsPLS1/VHA-A1* expression in the root, stem, young leaf, mature leaf, young panicle, and seeds. (B) Change over time in the *OsPLS1/VHA-A1* transcription level in the flag leaf. (C) *OsPLS1/VHA-A1* expression in different parts of the fully expanded leaf. (D) VHA activity. Values are means ±SD of four biological replicates. **P<0.01 (*t*-test).

To examine the temporal and spatial expression pattern of *OsPLS1/VHA-A1* in different tissues, a qRT-PCR assay was performed. As shown in Fig. 4A, the transcript levels of *OsPLS1/VHA-A1* varied remarkably in different genotypes and tissues. The wild type exhibited higher expression of *OsPLS1/VHA-A1* in the seeds, young leaves, stems, and mature leaves, in contrast to substantially lower expression in the same tissues of the *ospls1* mutant. Interestingly, the *OsPLS1/VHA-A1* gene was steadily and progressively down-regulated with natural age-dependent leaf senescence (Fig. 4B). From base to tip in the fully expanded leaf of both the *ospls1* mutant and its wild type, the mRNA levels of *OsPLS1/VHA-A1* trended to drop temporally (Fig. 4C). As expected, the VHA activity in the leaves of the *ospls1* mutant decreased by 85.02% in comparison with its wild type (Fig. 4D). These results suggested that the loss of *OsPLS1/VHA-A1* gene expression and VHA activity accelerated the age-dependent leaf senescence in *ospls1*.

### ROS accumulation and high SA levels, and enhanced signal transduction in *ospls1*

**Fig. 5.**
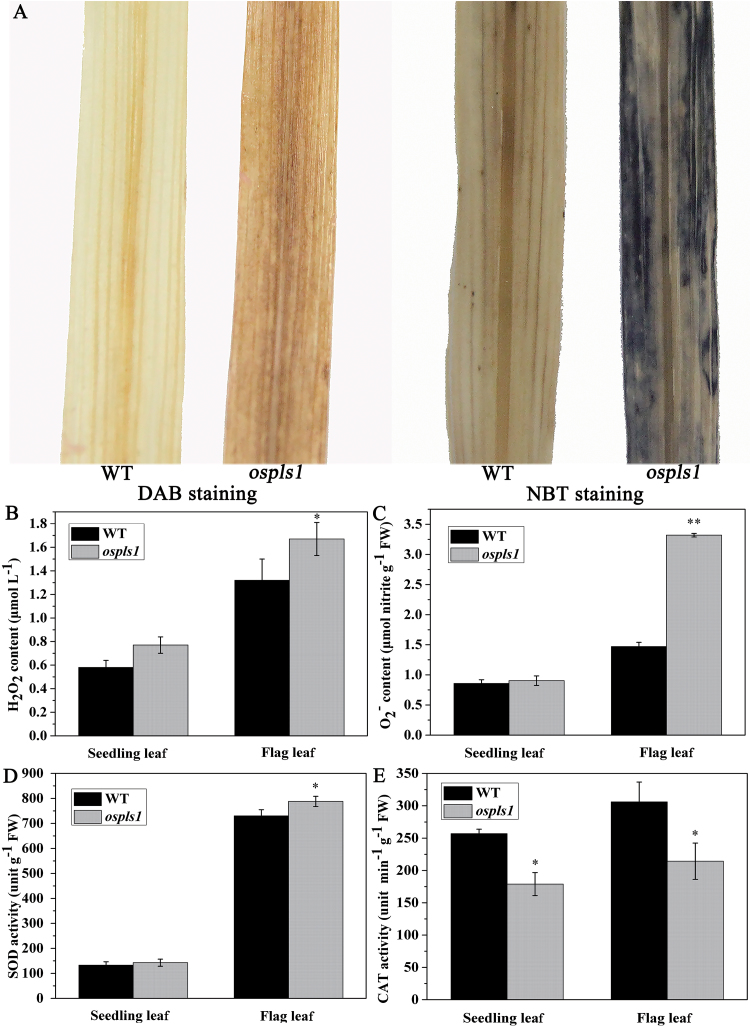
ROS accumulation and the activities of the antioxidant enzymes in *ospls1* and its wild-type leaf. Accumulation of H_2_O_2_ (A, B) and O_2_^–^ (A, C) was histochemically detected using NBT (A, for O_2_^–^) and DAB (A, for H_2_O_2_) or quantitatively measured (B, C). The activities of the antioxidant enzymes SOD (D) and CAT (E) in the leaf of the *ospls1* mutant and its wild type. Values are means ±SD of four biological replicates. *P<0.05, **P<0.01 (*t*-test).

The production of ROS is one of the earliest components of leaf senescence (Herrera-Vásquez *et al.*, 2015). This prompted us to investigate whether ROS accumulation occurred in *ospls1*. First, the accumulation of H_2_O_2_ and O_2_^–^ in the flag leaves was assessed by DAB and NBT staining, respectively. The mutant leaves displayed deeper staining than leaves from its wild type (Fig. 5A), indicating that the former had higher levels of ROS. The histochemical analysis was further supported by quantitative measurements of H_2_O_2_ and O_2_^–^ (Fig. 5B, C). The endogenous H_2_O_2_ levels in the *ospls1* mutant were 26.97% higher in the flag leaves and 33.51% higher in the seedling leaves, respectively, compared with its wild type (Fig. 5B). A similar result was observed with the O_2_^–^ level in the flag leaves, which was 125.85% higher in the *ospls1* mutant than in its wild type. However, there was no obvious difference in O_2_^–^ content between *ospls1* and its wild-type leaves at the seedling stage (Fig. 5C). Together, these results demonstrated that ROS accumulated in *ospls1*. Therefore, the senescence phenotype of the *ospls1* mutant was associated with ROS accumulation, which was ultimately connected to a null mutation of the *OsPLS1/VHA-A1* gene.

To control ROS levels and prevent their toxicity, plants synthesize antioxidative enzymes. In particular, SOD and CAT activities were examined. SOD activities in the *ospls1* mutant were 7.30% higher in the seedling leaves and 7.93% higher in the flag leaves than in its wild type (Fig. 5D). However, CAT activities in the *ospls1* plants were 30.37% lower in the seedling leaves and 29.93% lower in the flag leaves than in its wild type (Fig. 5E). The increase of SOD activity suggests that the *ospls1* mutant may actively respond to the O_2_^–^ accumulation and produce more H_2_O_2_, while the reduction of CAT activity will decrease scavenging of the additional H_2_O_2_, leading to its accumulation in *ospls1*.

**Fig. 6.**
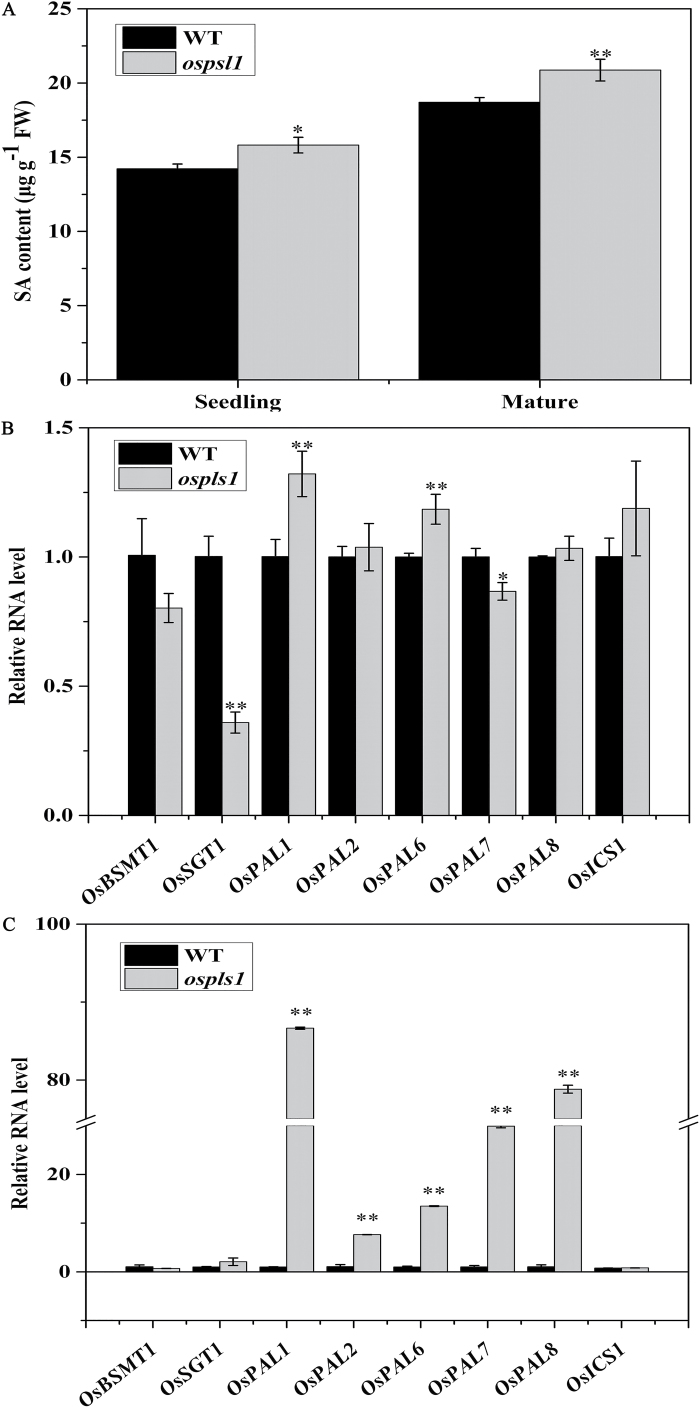
Effects on SA levels, and SA biosynthetic and metabolic genes in the *ospls1* mutant and its wild type. (A) Endogenous SA levels at the seedling and mature stage in the *ospls1* mutant and its wild type. (B, C) qRT-PCR analysis for SA biosynthetic and metabolic genes in leaf at the seedling stage and the mature stage in the *ospls1* mutant and its wild type. Values are means ±SD of four biological replicates. *P<0.05, **P<0.01 (*t*-test).

It has been demonstrated that ROS signals are involved in both upstream and downstream SA signaling in response to stress (Mori *et al.*, 2001; Herrera-Vásquez *et al.*, 2015). To explore whether ROS burst induced the SA level in the *ospls1* mutant, first we measured SA levels in leaves. The *ospls1* mutant had significantly higher SA levels than its wild type; they were 11.23% higher in mature leaves and 11.58% higher in seedling leaves, respectively (Fig. 6A). Next, we examined the expression of several genes involved in SA biosynthesis and metabolism. Relative to the wild type, mRNA expression of *OsSGT1* encoding an enzyme involved in SA metabolism was significantly lower, while mRNA levels of SA biosynthetic genes (*OsPAL2* and *OsPAL6*) were significantly higher in the *ospls1* mutant at the seedling stage (Fig. 6B). In addition, most of the genes responsible for SA biosynthesis, including *OsPAL1*, *OsPAL2*, *OsPAL6*, *OsPAL7*, and *OsPAL8*, showed remarkably higher expression in the *ospls1* mutant than in its wild type at the heading stage (Fig. 6C). Therefore, the phenotypes of the *ospls1* mutant were, at least in part, a result of the elevated SA levels, which were ultimately linked to a null mutation of the *OsPLS1/VHA-A1* gene.

**Fig. 7.**
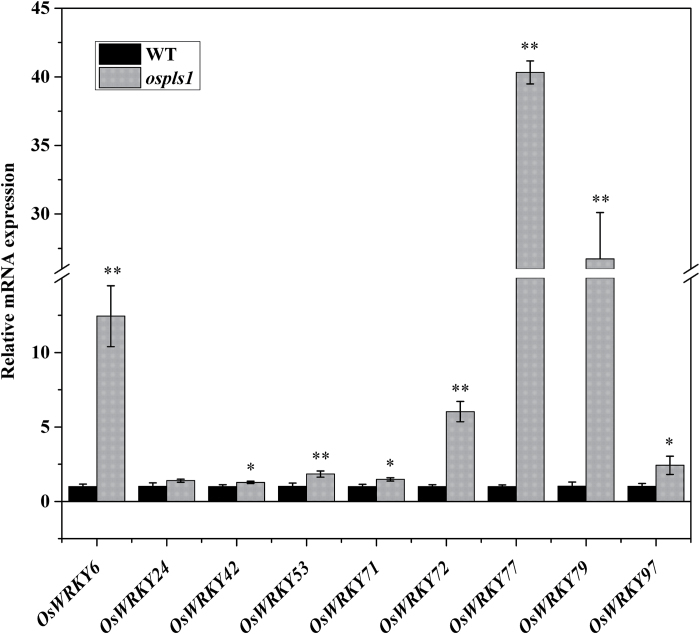
Analysis of *WRKY* gene expression in the *ospls1* mutant and its wild type. Values are means ±SD of four biological replicates. *P<0.05, **P<0.01 (*t*-test).

From previous studies by other groups (Zhou *et al.*, 2013; Bakshi and Oelmüller, 2014; Nuruzzaman *et al.*, 2014), SA and H_2_O_2_ are known to stimulate expression of *WRKY* genes, a group of important regulators for leaf senescence. As expected, a number of *WRKY* genes (*WRKY-6*, *-42*, -*53*, *-71*, *-72*, *-77*, *-79*, and *-97*) in the *ospls1* mutant displayed significantly higher expression than in its wild type (Fig. 7). These findings suggested important roles for *OsPLS1/VHA-A1* in the alteration of SA and H_2_O_2_ levels, and their signal transduction, which mediated leaf senescence in rice.

### Hypersensitivity to exogenous H_2_O_2_ and SA

**Fig. 8.**
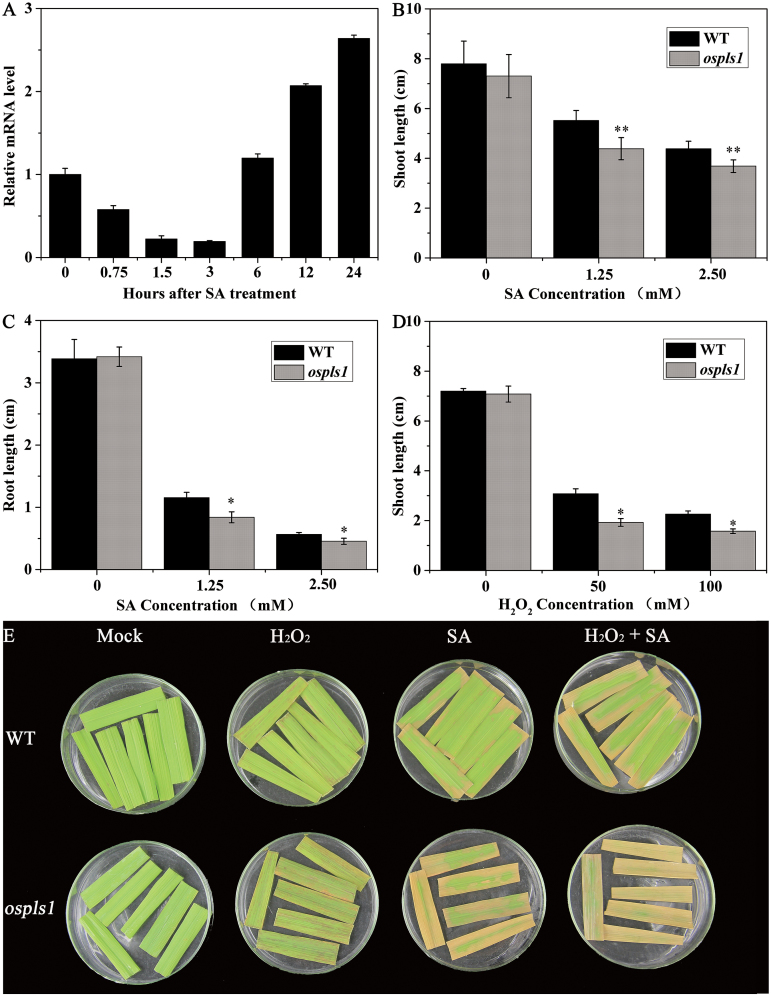
The *ospls1* mutant response to exogenous SA and/or H_2_O_2_. (A) Effects of exogenous SA on *OsPLS1/VHA-A1* expression. Shoot (B) and primary root (C) lengths of the seedlings grown for 6 d on 1/2 MS plates without (mock) or with different SA concentrations. (D) Shoot lengths of the seedlings grown for 6 d on 1/2 MS plates without (mock) or with different H_2_O_2_ concentrations. Error bars indicate the SD (*n*=30). *P<0.05, **P<0.01 (*t*-test). (E) Detached flag leaves at the primary heading stage were mock treated or treated with 5mM SA and/or 100mM H_2_O_2_ for 2.5 d in darkness.

The participation of the *OsPLS1/VHA-A1* gene in leaf senescence mediated by H_2_O_2_ and SA signals could be further supported by exogenous SA and/or H_2_O_2_ treatments. As shown in Fig. 8A, the transcripts of the *OsPLS1/VHA-A1* gene exhibited a two-phase pattern for the three- to four-leaf seedlings exposed to SA solution; they were suppressed during the initial 3h, and then were restored and gradually enhanced (Fig. 8A). This observation demonstrated a direct regulation of *OsPLS1/VHA-A1* by SA. To determine the impact of exogenous SA or H_2_O_2_ on seedling growth, the *ospls1* mutant and its wild type were grown for 6 d in 1/2 MS medium supplemented with SA or H_2_O_2_. Under normal conditions, no visible difference in the shoot and root length was observed between the *ospls1* mutant and its wild type. However, the shoot and root of the *ospls1* mutant were significantly shorter than those of its wild type when SA was present in the medium, despite a slightly retarded development for the wild type (Fig. 8B, C; Supplementary Fig. S3A). Similar results were also found under H_2_O_2_ treatment; the *ospls1* mutant displayed more sensitivity than its wild type (Fig. 8D; Supplementary Fig. S3B).

Next, the fully expanded flag leaves were detached for mock treatment or treatment with 5mM SA and/or 100mM H_2_O_2_ for 2.5 d in darkness. With mock treatment, the *ospls1* mutant leaves, similar to its wild-type leaves, showed no senescence phenotype. Under separate and combined treatment of SA and H_2_O_2_, the *ospls1* mutant had more exacerbated leaf senescence than its wild type. However, the *ospls1* mutant was slightly sensitive to H_2_O_2_, and was hypersensitive to SA with or without H_2_O_2_. Furthermore, the combined effect of SA and H_2_O_2_ on leaf senescence was the most intense (Fig. 8E). These results suggested that SA probably had the critical role and interplayed with ROS for regulation of leaf senescence in *ospls1*.

### Seed dormancy of the *ospls1* mutant grains

**Fig. 9.**
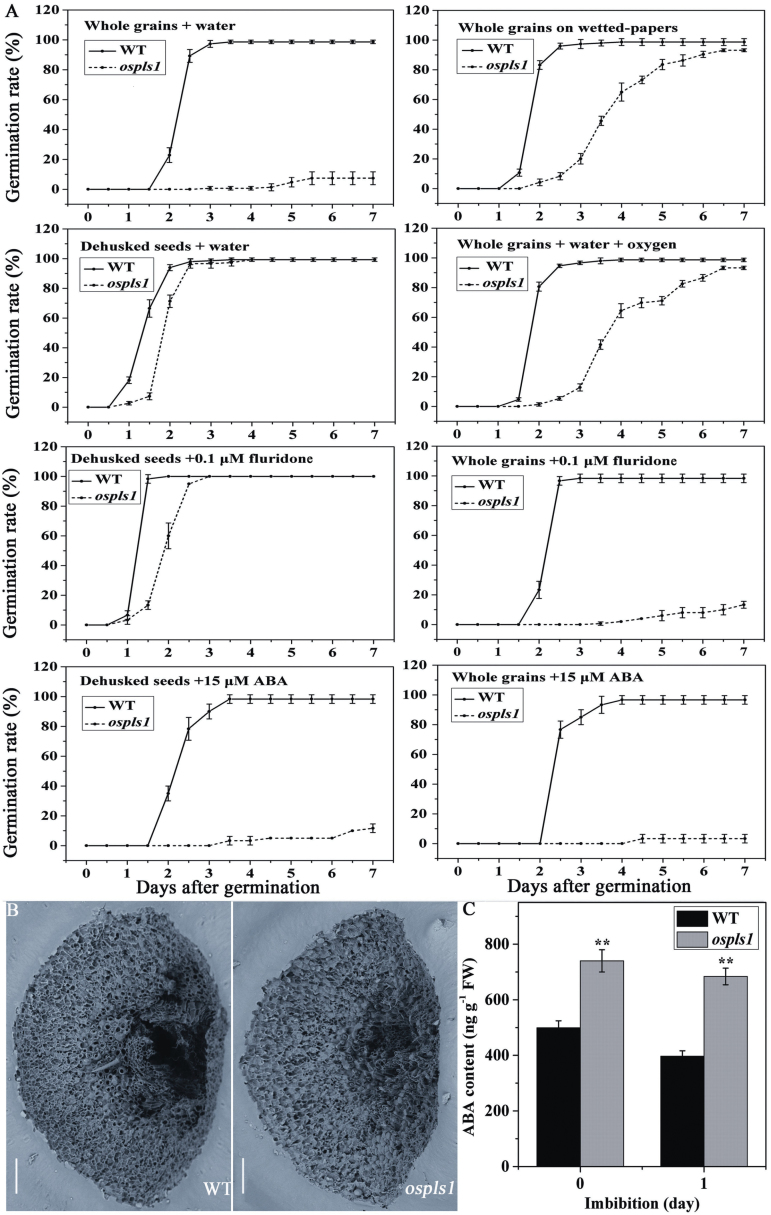
Germination analysis, SEM observation, and ABA contents of the *ospls1* mutant seeds. (A) Germination rates of the mature grains under various conditions. Values are means ±SD of four biological replicates. (B) SEM observation of the micropyle of the *ospls1* mutant and its wild-type seeds. Scale bar=150 μm. (C) ABA levels in dehulled seeds of the *ospls1* mutant during imbibition. Values are means ±SD of four biological replicates. **P<0.01 (*t*-test).

Previous studies indicated that VHA subunits were implicated in grain development and germination (Cooley *et al.*, 1999; Amemiya *et al.*, 2006). To explore the potential effect of the *OsPLS1/VHA-A1* mutation on grain structure and germination, germination rates of the mature grains were assayed under various conditions (Fig. 9A). First, under complete submergence in water, intact grains of the *ospls1* mutant showed only a 7% germination rate, whereas those of the wild type reached 98%. Moreover, we also tested intact grains from the six pOsPLS1 transgenic lines (Fig. 3I) and found that their germination rates were the same as those of the wild type. Therefore, the transgenic introduction of wild-type *OsPLS1/VHA1* in the *ospls1* mutant restored its germination. Taken together, the *OsPLS1/VHA1* mutation resulted in seed dormancy or seed vigor in the *ospls1* mutant.

Considering the high efficiency of glumellae removal in breaking dormancy in rice (Waheed *et al.*, 2012), we employed dehulled grains to monitor the germination (Fig. 9A). Interestingly, the final germination rate of dehulled grains of the *ospls1* mutant was similar to that of its wild type, with nearly 100% under the same conditions as described above (Fig. 9A), arguing that the glumellae were an important factor for seed dormancy in the *ospls1* mutant. We hypothesized that there was a structural abnormality in the glumellae of the *ospls1* mutant. SEM observation demonstrated that the *ospls1* mutant had shallow and compact micropyles, clearly distinct from those of its wild type (Fig. 9B). It has been proposed that the effect of the micropyle on dormancy is to reduce oxygen penetration into the embryo (Bradford *et al.*, 2008). To test whether the micropyles of the *ospls1* mutant inhibit oxygen uptake, the intact grains were simultaneously germinated on wetted papers. Compared with the wild type, the *ospls1* mutant achieved a final 93% germination rate and also displayed slower germination (Fig. 9A), suggesting that oxygen is an important factor for breaking dormancy of the *ospls1* mutant. Consistent with this observation, the *ospls1* mutant also achieved a similarly high germination rate under complete submergence supplemented with air-pumped oxygen (Fig. 9A). These findings suggested that mutation of *OsPLS1/OsVHA1* mediated seed dormancy partially due to a developmental defect of the micropyle, which limited oxygen penetration under submergence in the *ospls1* mutant.

### Sensitivity of seed germination to ABA in *ospls1*

It is recognized that hypoxia interferes with ABA metabolism, and ABA is believed to be a central player in establishment and maintenance of seed dormancy (Benech-Arnold *et al.*, 2006; Bradford *et al.*, 2008). To determine whether ABA levels correlated with germination, we measured ABA amounts in dehulled seeds germinated under submergence. The *ospls1* mutant grains had significantly higher ABA levels than those of its wild type (Fig. 9C), at 48.12% and 72.28% higher levels in the dehulled grains before and after 1 d of incubation, respectively. Moreover, the changes in ABA contents during incubation were also different between *ospls1* and the wild type: ABA levels in the wild-type dehulled grains decreased more sharply than in *ospls1* (Fig. 9C). In addition, to test whether hormones, in particular ABA and GA_3_, affected the germination, the intact or dehulled grains were germinated in aqueous solutions of GA_3_, ABA, or fluridone. Interestingly, despite their slower germination, nearly 100% intact and dehulled grains of the wild type were able to germinate in a range of ABA solutions (Fig. 9A; Supplementary Fig. S4). In contrast, in the presence of >15 µM ABA solution, the *ospls1* dehulled grains showed an ~10% germination rate, while the germination of the intact grains was suppressed completely (Fig. 9A). To evaluate further the contribution of ABA biosynthesis or metabolism to dormancy during seed imbibition, grains were germinated in the presence of the herbicide fluridone, which interferes with ABA biosynthesis due to blocking its carotenoid precursors (Matusova *et al.*, 2005). Fluridone did not clearly stimulate germination of the intact and dehulled grains of the mutant as compared with the control (Fig.9A). These results suggested that ABA catabolism during seed imbibition accounted for germination retardation to a certain extent in the *ospls1* mutant. As GA_3_ functions antagonistically with ABA in controlling germination and dormancy, to investigate its effects on germination, grains were germinated under complete submergence in GA_3_ solution with or without fluridone. Despite the fact that it slightly acceleraed germination, GA_3_ with or without fluridone did not improve the germination of the mutants as compared with the control (Supplementary Fig. S4). Taken together, these results indicated that *OsPLS1/OsVHA1* might be involved in ABA signaling, in particular ABA metabolism, which played a critical role in regulating seed dormancy of the *ospls1* mutant.

## Discussion

VHA-A is a highly evolutionarily conserved enzyme complex. A series of investigations have been reported regarding its diverse functions in different developmental processes including conditional lethality in the *tfp1-∆8* mutant (Kane *et al.*, 1990), and complete male and partial female gametophtic lethality in the *Arabidopsis vha-A* mutant (Dettmer *et al.*, 2005). These studies suggest that the *VHA-A* gene is involved in regulation of cell growth and death, but it is unclear whether *VHA-A* participates directly in leaf senescence. However, the findings in our study support an important role for *OsPLS1/VHA-A* in leaf senescence. In the present study, we reported the identification of an *ospls1* mutant characterized by an accelerated lesion-mimic and early senescence phenotype (Fig. 1A, F), and crop yield reduction (Supplementary Table S4). Based on fine-mapping, we found a cytosine deletion at the 312^th^ nucleotide of the *OsPLS1/VHA-A1* gene in the *ospls1* mutant. Transgenic expression of wild-type *OsPLS1/VHA-A1* can recover the phenotypic abnormalities in the *ospls1* mutant (Fig. 3I). Of note, the expression levels of *OsPLS1/VHA-A1* were down-regulated during natural age-dependent leaf senescence in both the *ospls1* mutant and its wild type (Fig. 4B). Consistent with this temporal observation, a progressive decrease in *OsPLS1/VHA-A1* expression was also observed from the base to the tip of a fully expanded leaf in both the *ospls1* mutant and its wild type. These results revealed a negative correlation between *OsPLS1/VHA-A1* gene expression and leaf senescence. A cytosine deletion in the *OsPLS1/VHA-A1* gene in the *ospls1* mutant led to its lower gene expression in all tissues, and much lower VHA activity in fully expanded flag leaves as compared with the wild type. These results further proved that higher expression of *OsPLS1/VHA-A1* or high activity of VHA was required for a delay of the lesion-mimic and senescence phenotype in rice. To our knowledge, this is the first identification of the role of *OsPLS1/VHA-A1* in leaf senescence.

Leaf senescence is the final stage of leaf development and is controlled by various internal and external factors (Lim *et al.*, 2007; Zhou and Gan, 2009). Generation of ROS is one of the earliest responses of plant cells under abiotic stresses and senescence (Herrera-Vásquez *et al.*, 2015). Zhang *et al.* (2015) revealed that increasing the duration and intensity of the dehydration stress resulted in ROS accumulation due to high stomatal density in wild-type trifoliate orange as compared with the transgenic lines overexpressing *PtrABF*. In this study, it is demonstrated that null mutation of *OsPLS1/VHA-A1* resulted in a high stomatal density (Fig. 2), transpiration rate (Fig. 1K), and increasing water loss in *ospls1* (Supplementary Fig. S2). These in turn conferred enhanced dehydration sensitivity and ROS accumulation (Fig. 5) in *ospls1.* In addition, ROS-mediated chloroplast degradation occurs during leaf senescence (Khanna-Chopra, 2012). Thus, ROS accumulation is the most likely reason for the grana breakdown in *ospls1* (Fig. 1D, E). These results indicated that ROS may play an important role in the leaf senescence of *ospls1*.

SA is another prominent endogenous factor that induces leaf senescence through inhibiting photosynthetic electron transport (Janda *et al.*, 2012), and causes destruction of the thylakoid (Uzunova and Popova, 2000). Our finding of a two-phase regulation of *OsPLS1/VHA-A1* expression by exogenous SA treatment (Fig. 6A) suggested that *OsPLS1/VHA-A1* mutation resulted in an alteration in the SA signal pathway in rice. Tyagi *et al.* (2005) proposed the presence of an SA response element (TCA) in the *PgVHA-c1* promoter, and a 2-fold up-regulation of *PgVHA-c1* expression was induced in 15-day-old *Pennisetum* shoots exposed to 50 μM exogenous SA for 24h. The sequence analysis also showed that there were two TCA elements in the *OsPLS1/VHA-A1* promoter region (data not shown). This observation illustrated that *OsPLS1/VHA-A1* was closely associated with SA signaling.

To date, the crosstalk between *OsPLS1/VHA-A1* and the SA signaling pathway for the regulation of leaf senescence is poorly understood. The endogenous SA levels increased during leaf senescence in Arabidopsis (Morris *et al.*, 2000), and SA accumulation showed a positive correlation with production of ROS in plants (Mori *et al.*, 2001; Herrera-Vásquez *et al.*, 2015). In the current study, the *ospls1* mutant exhibited higher accumulation of endogenous SA (Fig. 6A), which appeared to be a factor promoting acceleration of senescence and enhancing expression of senescence-associated genes (SAGs) in the leaves (Fig. 1H–J). Higher accumulation of endogenous SA appeared with ROS accumulation (Fig.5A–C), and up-regulation of SA biosynthesis genes was found in the *ospls1* mutant (Fig. 6B, C). On the other hand, the expression of the SA metabolism gene *OsSGT1*, a SA glucosyltransferase for catalyzing the conversion of free SA into SA 2-*O*-β-d-glucoside, was inhibited by >2-fold in the *ospls1* mutant as compared with the wild type at the seedling stage (Fig. 6B). Down-regulation of this gene would decrease SA conjugation with glucose and result in accumulation of free SA in the *ospls1* mutant (Fig. 6A). A previous study with a tobacco suspension cell culture showed that SA and SA 2-*O*-β-d-glucoside could be transported across the tonoplast, and stored in the vacuoles (Dean *et al.*, 2005). Therefore, pharmacological inhibition of VHA activity by baﬁlomycin A1 decreased the uptake of ATP-dependent SA 2-*O*-β-d-glucoside by 80% in tobacco cells (Dean *et al.*, 2005). In the *ospls1* mutant, the VHA activity was significantly decreased because of down-regulation of *OSPLS1/VHA-A1* expression (Fig. 4D). Thus, the potentially impaired uptake of SA and SA 2-*O*-β-d-glucoside into the vacuoles would similarly lead to SA accumulation in the cytoplasm of the *ospls1* mutant due to its lower VHA activity. Further investigations are needed to confirm the reduction of SA and SA 2-*O*-β-d-glucoside in the vacuoles of the *ospls1* mutant.

Altered SA and/or H_2_O_2_ signal transduction can impact senescence phenotypes. The expression of many *WRKY* genes was induced by SA and/or H_2_O_2_, supporting the involvement of *WRKY* transcription factors in leaf senescence (Bakshi and Oelmüller, 2014). Overexpression of *OsWRKY42* has been shown to cause leaf senescence by repressing *OsMT1d* expression in rice (Han *et al.*, 2014). In the present study, the mRNA levels of *WRKY* genes were significantly higher in the *ospls1* mutant than in its wild type (Fig. 7), suggesting that SA and/or H_2_O_2_ signal transduction was enhanced in *ospls1*. In addition, SA or H_2_O_2_ inhibition was significantly higher in the *ospls1* seedlings than in its wild type, suggesting that the *ospls1* mutant was hypersensitive to the combination of exogenous SA and H_2_O_2_ (Fig. 8B–D; Supplementary Fig. S3). These results further indicate that the interplay between SA and ROS is proposed in the regulation of the *OsPLS1/VHA-A1*-mediated leaf senescence.

Besides its impact on lesion-mimic and premature leaf senescence, the *ospls1* mutant also showed high susceptibility to drought and salt stress as expected (data not shown) (Zhang *et al.*, 2013), and unexpected seed dormancy. The micropyle, the most important part of the glumella, provides an entrance channel for oxygen into the embryo for activation of germination. The inhibition of seed germination by the micropyle and a wide variation of mean dormancy periods have been observed in rice (Roberts, 1961). In the present study, the germination rate of intact grains of the *ospls1* mutant under complete submergence is significantly lower than that of its wild type, while the final germination rate of the dehulled grains of the *ospls1* mutant was similar to that of its wild type under the same conditions (Fig. 9A). Moreover, at 90 d of after-ripening, the intact grains of the *ospls1* mutant still maintained very low germination (data not shown). However, the dehulled grains of the *ospls1* mutant displayed slower germination and ABA metabolism, and higher ABA levels as compared with its wild type (Fig. 9A, C). Taken together, the present results confirmed the importance of glumellae, in particular the micropyle structure, in seed dormancy of the *ospls1* mutant, but did not support the micropyle as the key factor in the physiological maintenance of dormancy. Indeed, glumellae appear to mediate dormancy in concert with other endogenous constituents including ABA and GA_3_ (Benech-Arnold *et al.*, 2006; Gianinetti and Vernieri, 2007; Bradford *et al.*, 2008). Involvement of grain responsiveness to ABA in the regulation of seed dormancy has been reported in barley (Benech-Arnold *et al.*, 2006; Bradford *et al.*, 2008) and red rice (Gianinetti and Vernieri, 2007). In this study, it was clearly demonstrated that the *ospls1* mutant grains were more sensitive to exogenous ABA than those of its wild type (Fig. 9A; Supplementary Fig. S4). In addition, fluridone significantly inhibited endogenous ABA synthesis and broke seed dormancy in red rice (Gianinetti and Vernieri, 2007). Thus, the response of the *ospls1* mutant grains to fluridone solution suggested that the mutant seeds were in a dormant state predisposed to the action of ABA metabolism, and not of ABA synthesis (Fig. 9A). Seed development has been shown to be correlated with the *VHA-A* expression level in that *LeVHA-A*-suppressed plants had smaller fruits with fewer seeds relative to the control (Amemiya *et al.*, 2006). Seed coat-forming cells of normal grains developed prominent tannin vacuoles which persisted throughout grain development (Felker *et al.*, 1984). On the other hand, ABA conjugated with glucose can be transported and stored in the vacuoles (Dong and Hwang, 2014). Presumably, a null mutation of *OsPLS1/VHA-A1*, which is localized in the vacuolar membranes (Zhang *et al.*, 2013), may have impaired mostly the vacuolar function in micropyle structure and ABA signal in *ospls1*. Hence, these results indicated that *OsPLS1/VHA-A1* participated in the development of the micropyle and ABA signaling, and mutation in *OsPLS1/OsVHA-A1* impaired seed germination.

However, crosstalk between the micropyle and ABA, which mediates seed dormancy in *ospls1*, remains to be determined. The restriction of germination by the glumella has been attributed to its ability to reduce oxygen penetration into the embryo (Bradford *et al.*, 2008). Under hypoxic conditions, dormant barley grains displayed greater sensitivity to ABA and interference with ABA metabolism as compared with non-dormant grains (Benech-Arnold *et al.*, 2006). In general, the concentration of oxygen in air and distilled water at 25 °C is 21% and 0.52%, respectively. In this study, only 7% of intact grains of the *ospls1* mutant germinated in water. It is possible that the micropyle limits oxygen penetration into the embryo during flooding, and lower oxygen results in maintenance of high ABA contents (Fig. 9C), which will limit weakening of the structures surrounding the embryo, cell wall loosening, and radicle extension (Bewley, 1997). In fact, the intact grains of the *ospls1* mutant can be germinated almost completely on wetted paper (Fig. 9A), suggesting that enough oxygen could also promote ABA metabolism and break the seed dormancy of the *ospls1* mutant (Bradford *et al.*, 2008). However, how OsPLS1/VHA-A1 regulates ABA catabolism remains to be investigated.

To summarize, we present the first report on the requirement for *OsPLS1*/*VHA-A1* in premature leaf senescence and seed dormancy. We have identified a single cytosine deletion in the *OsPLS1/VHA-A1* gene encoding VHA-A1. ROS accumulation, higher endogenous SA levels, and more active ROS and/or SA signal transduction substantiated by higher expression of *WRKY* factors were concomitantly detected in the *ospls1* mutant. Moreover, the *ospls1* mutant showed severe seed dormancy. Further studies are needed to elucidate the molecular mechanism underlying *OsPLS1/VHA-A1*-mediated leaf senescence by ROS and/or the SA signaling pathway; it will be helpful to understand fully why *OsPLS1/VHA-A1* mutation leads to early senescence, the role of *OsPLS1/VHA-A1* in SA 2-*O*-β-d-glucoside transport across the tonoplast, ROS and/or SA induction of leaf senescence, and seed dormancy due to ABA signaling and inhibition of oxygen uptake in rice.

## Supplementary data

Supplementary data are available at *JXB* online.

Table S1. List of molecular markers for fine-mapping of *OsPLS1.*

Table S2. Primers for detection of the mutation site, construction of the functional complementary vector, and confirmation of positive transgenic rice.

Table S3. List of primers used for qRT-PCR analysis.

Table S4. Main agronomic traits of the *ospls1* mutant and its wild type.

Table S5. Analysis of various gas exchange parameters in the *ospls1* mutant and its wild type.

Table S6. Segregation of F_2_ populations from two crosses.

Figure S1. Phenotype of the *ospls1* mutant and its wild type at the heading stage.

Figure S2. Rates of water loss from the detached leaves of the *ospls1* mutant and its wild type.

Figure S3. Phenotypes of the *ospls1* mutant and its wild-type response to exogenous SA or H_2_O_2_.

Figure S4. Germination analysis of the *ospls1* mutant and its wild-type seeds under ABA and GA_3_ with and without fluridone.

Supplementary Data
