# Supplementary material for: A single cytosine deletion in the OsPLS1 gene encoding vacuolar-type H+-ATPase subunit A1 leads to premature leaf senescence and seed dormancy in rice
Source: J Exp Bot. 2016 Mar 19;67(9):2761–76. doi: 10.1093/jxb/erw109 (PMC4861022; doi:10.1093/jxb/erw109)
Supplement: Supplementary Data [file supp_67_9_2761__index.html]

A single cytosine deletion in the OsPLS1 gene encoding vacuolar-type H+-ATPase subunit A1 leads to premature leaf senescence and seed dormancy in rice — A single cytosine deletion in the OsPLS1 gene encoding vacuolar-type H+-ATPase subunit A1 leads to premature leaf senescence and seed dormancy in rice — Supplementary Data 

# A single cytosine deletion in the *OsPLS1* gene encoding vacuolar-type H+-ATPase subunit A1 leads to premature leaf senescence and seed dormancy in rice

## Supplementary Data

Data files

- supplementary\_tables\_S1\_S6\_figures\_S1\_S5.pdf - Supplementary Data
