# Supplementary material for: A single cytosine deletion in the OsPLS1 gene encoding vacuolar-type H+-ATPase subunit A1 leads to premature leaf senescence and seed dormancy in rice
Source: J Exp Bot. 2016 Mar 19;67(9):2761–76. doi: 10.1093/jxb/erw109 (PMC4861022; doi:10.1093/jxb/erw109)
Supplement: Supplementary Data [file supp_erw109_supplementary_tables_S1_S6_figures_S1_S5.pdf]

Supporting information

Additional supporting information may be found in the online version of this article

Table S1 List of molecular markers for fine mapping of *OsPLS3*

Table S2 Primers for detection of mutation site, construction of functional complementary vector and confirmation of positive transgenic rice

Table S3 List of primers used for q-RT PCR analysis

Table S4 Main agronomic traits of the *ospls1* mutant and its wild-type

Table S5 Analysis of various gas exchange parameters in the *ospls1* mutant and its wild-type

Table S6 Segregation of F_2 populations from two crosses

Figure S1 Phenotype of the *ospls1* mutant and its wild-type at the heading stage.

Figure S2 Chloroplast ultrastructure of the *ospls1* mutant and its wild-type.

Figure S3 Rates of water loss from the detached leaves of the *ospls1* mutant and its wild-type.

Figure S4 Phenotypes of the *ospls1* mutant and its wild-type response to exogenous SA or H_2O_2.

Figure S5 Germination analysis of the *ospls1* mutant and its wild-type seeds under ABA, and GA_3 with and without fluridone.

Figure S legends

Figure S1 Phenotypes of the *ospls1* mutant and its wild-type at the heading stage. Phenotypes of whole plants (A) and leaves (B). F means flag leaf; 2-4 mean the 2nd to the 4th leaf from top.

Figure S2 Chloroplast ultrastructure of the *ospls1* mutant and its wild-type. (A-B) wild-type plants. (C-D) the *ospls1* mutant. C, chloroplast; CW, cell wall; M, mitochondrion; N, nucleus; OS, osmiophilic body; S, starch grain; Th, thylakoid; PD, plasmodesma.

Figure S3 Rates of water loss from the detached flag leaves of the *ospls1* mutant and its wild-type during the dehydration treatment, measured every 1 h. Error bars indicate the SD (n=4).

Figure S4 Phenotypes of the *ospls1* mutant and its wild-type response to exogenous SA (A) or H_2O_2 (B).

Figure S5 Germination analysis of the *ospls1* mutant and its wild-type seeds under ABA, and GA_3 with and without fluridone.

Table S1 List of molecular markers for fine mapping of *OsPLS3*

Maker name	Forward sequence (5’-3’)	Reverse sequence (5’-3’)
S1	GAGGCTTGAGGTTACTATGGA	CGGAGAGGTTAGCAGTGTAGT
S2	GCTATGACAGATCGCTTATCC	TCAGATCGTGAACTCAAGTGT
S3	ATCGATCAACACAACACACAT	GAGATTCTCTGCCGTTGC
S4	TATGTGTTTTCTGGGTGAATG	CATCTTACTGACGCAATGAAC
S5	GGGAGAGAGCAGCTAAGAATA	AACAAGAAAACGTTGCAGTCT

Table S2 Primers for detection of mutation site, construction of functional complementary vector and confirmation of positive transgenic rice

Primer name	Forward sequence (5’-3’)	Reverse sequence (5’-3’)
CAPS-PLS1	CTTTCTACACTTTGCAGGGCC	CAGGCACAATACCTAGCTTC
PLS1-promoter	CCAAGCTTGCATGCCGGAAACTGTGGTCACAAAAT	CGTACTCGCTCTCCTTCTC
PLS1-cDNA	CATGTCGTACGATCGCGTCAC	CCTCTAGAGTCGACCTCACCTAGCTTCATCTTCTAG
PLS1	AGGAGAGCACTATGAATGAGG	AATGATGATGGCTTCTTTCTC
HPH	GTAAATAGCTGCGCCGATGG	TACTTCTACACAGCCATCGG

Table S3 List of primers for qRT PCR analysis

Gene name	Forward sequence (5’-3’)	Reverse sequence (5’-3’)
UBQ10	TGGTCAGTAATCAGCCAGTTTGG	GCACCACAAATACTTGACGAACAG
OsPLS1	GCTAACACATCCAACATGCC	TTATAGCCCATGTCACGGAA
RCCR1	CGCATTTCCTCATGGAATTT	CTTCTCACGCTGTTTGTCCA
SGR	AGGGGTGGTACAACAAGCTG	GCTCCTTGCGGAAGATGTAG
OsI57	ACCCTAAAGTAAATGAAGTC	CCTGCTCTTGTCTTGTTA
OsBSMT1	TCAAGAGAAGGCCATCCTGAA	AGAGCGACGCGTGAGCTT
OsSGT1	AGGTGTGTGAGGGAGGTGAT	CATTTCTGGCCTTCTCCTTC
OsPAL1	AGGAGCTCGGCTGCGTATT	ATGCCGAGGAACACCTTGTT
OsPAL2	AGCTGGTCAACGAGTTCTACAACA	GAGGGAGTTGACGTCCTGGTT
OsPAL6	GGGCAACCCAGTGACCAA	CGATTGCCTCGTCGGTCTT
OsPAL7	CCAACCCTGTGACCAACCAT	GATCAAGAACGTCGAGGACATG
OsPAL8	GCTTCTTCGAGTTGCAGCCTAA	CAGGACCTCGGCGAGGAT
OsICS1	TATGGTGCTATCCGCTTCGAT	CGAGAACCGAGCTCTCTTCAA
OsWRKY6	ATTGTGTTTGGGTTTGATGC	ACACCTCTCGACGTGCTTC
OsWRKY24	CGCTTCCTCCTTCTTCCC	GTGCGGTTCGTGTCTCTGT
OsWRKY42	CAATTGACCGTTTGAATATTGG	TGAGGGAGGTAGCAGATCAA
OsWRKY53	TAGCTCGCTTGCTTGCTG	GTGAACGACGTGATGAACG
OsWRKY71	CTCAGCTCCTCCTCTCACCT	ATCACTGATCCGACGACCTA
OsWRKY72	GGTCGAGATGGAGAACTTCC	GGAGCTGGAGGTAGAAGACG
OsWRKY77	AGGTGATCGCTCTCTTCAGG	GCCCAGGACTCACACTAGC
OsWRKY79	TCCCAGTCTGTGTGTGAGTG	GCTTAGCCAATTACCAACCAA
OsWRKY97	ATCGCTTTGCTTGATACCCT	TCGACTTTCTTCTCGTCCCT

Table S4 Main agronomic traits of the *ospls1* mutant and its wild-type

Traits	Wild-type	*ospls1*
Plant height (cm)	103.25 ±2.55	74.28 ±3.13**
Effective panicle number	7.78 ±0.85	6.61 ±0.78
Panicle length (cm)	24.37 ±0.88	21.09 ±1.24**
Grain number per panicle	149.63 ±9.32	112.35 ±11.02**
Seed setting rate (%)	82.24 ±9.30	38.64 ±4.11**
1000-grain weight (g)	22.76 ±0.84	18.95 ±1.12**
Yield per plant (g)	22.89 ±3.51	5.43 ±0.89**

** Significantly different at P<0.01 (t-test).

Table S5 Analysis of various gas exchange parameters in the *ospls1* mutant and its wild-type

Parameters	Wild-type	*ospls1*
Photosynthetic efficiency (μ mol CO_2 /m^2s)	18.30±0.62	18.70±0.35
Stomatal conductance (μ mol H_2O/ m^2s)	1.01±0.017	1.05±0.064
Intercellular CO_2 concentration (μ mol CO_{2}/ mol)	329.00±2.52	324.33±3.21
Transpiration rate (μ mol H_2O/ m^2s)	7.18±0.16	8.00±0.070**

** Significantly different at P<0.01 (t-test).

Table S6 Segregation of F2 populations from two crosses

Cross	Wild-type	Early senescence mutant	Total	χ^2(3:1)	P value
ospls1/N142	525	173	698	0.0076	0.93053
ospls1/02428	961	315	1276	0.051	0.821332

Note: Note：The female/male parents are showed in the cross. $\chi^2 < \chi^2_{0.05}$=3.84 is for 3:1 segregation ratio. P>0.05 is considered as significant.

Fig. S1

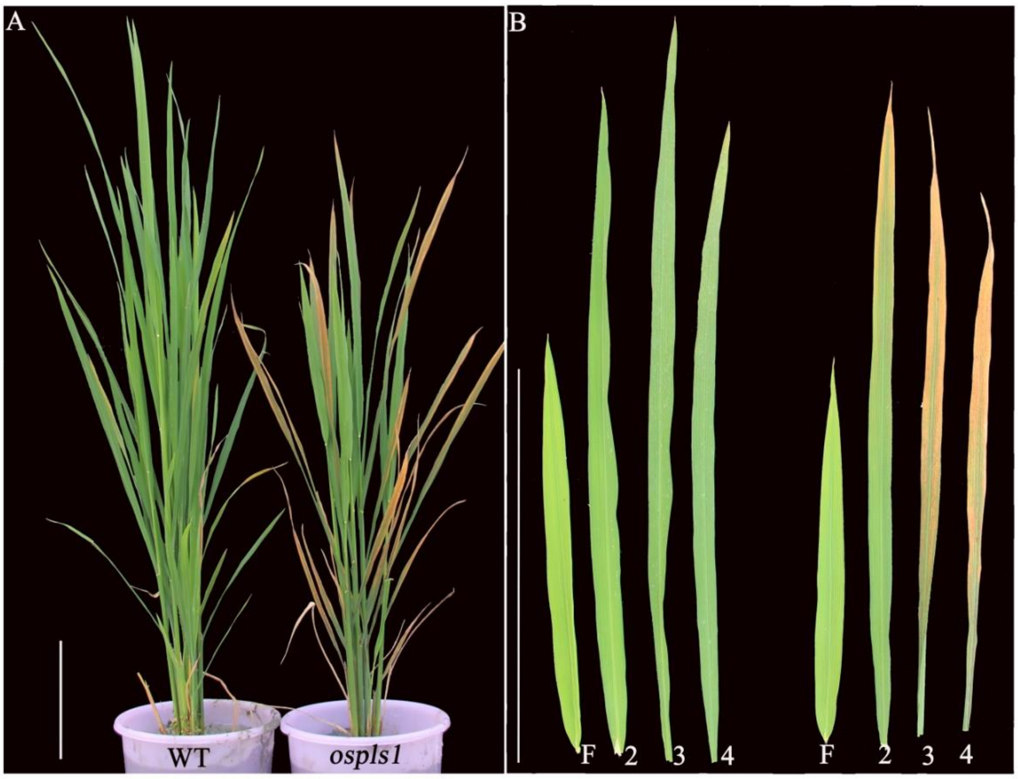

Fig. S2

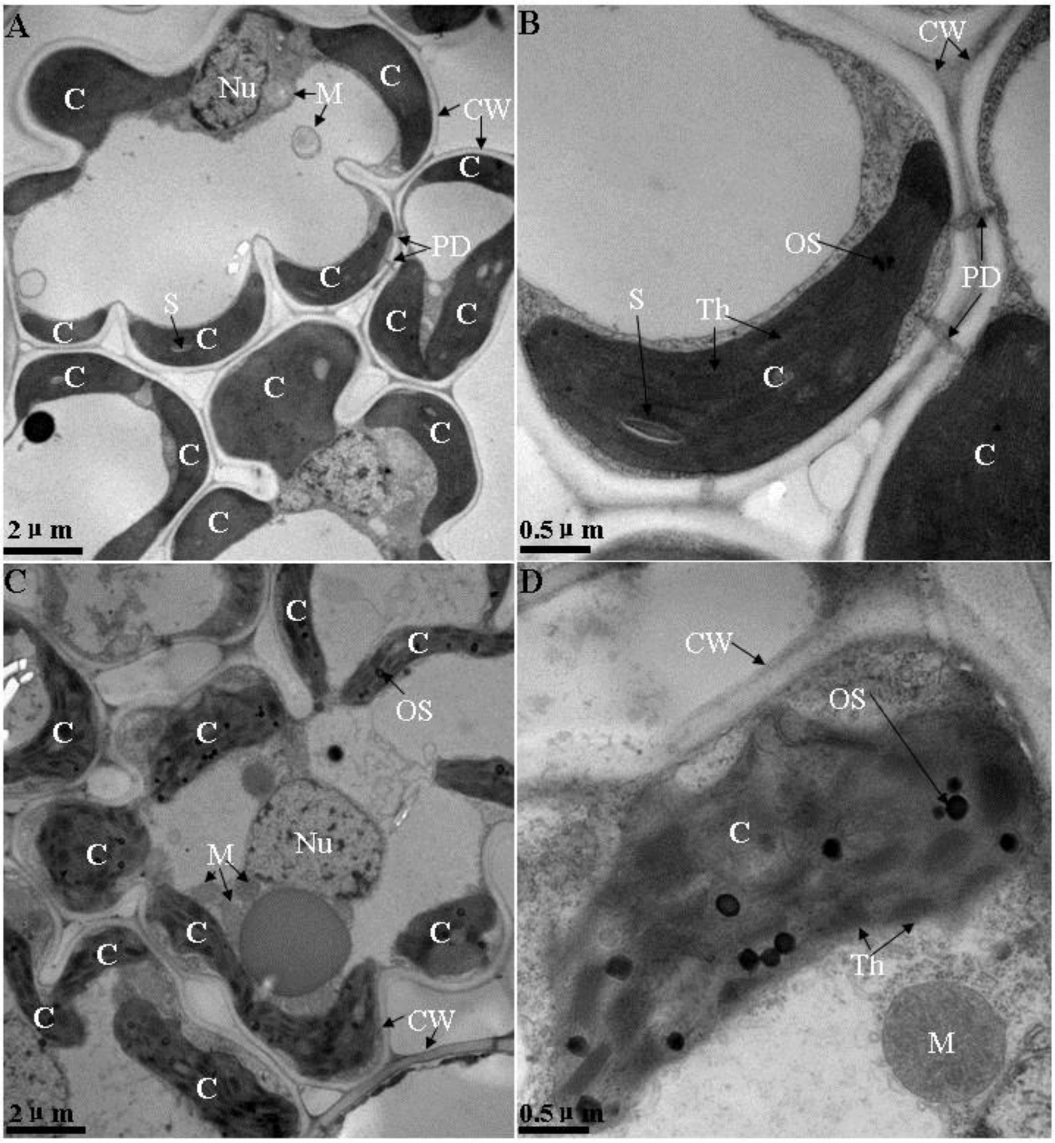
A
Nu
M
C
CW
PD
S
2 μ m
B
CW
OS
PD
S
Th
0.5 μ m
C
OS
Nu
M
CW
2 μ m
D
CW
OS
Th
M
0.5 μ m

Fig. S3

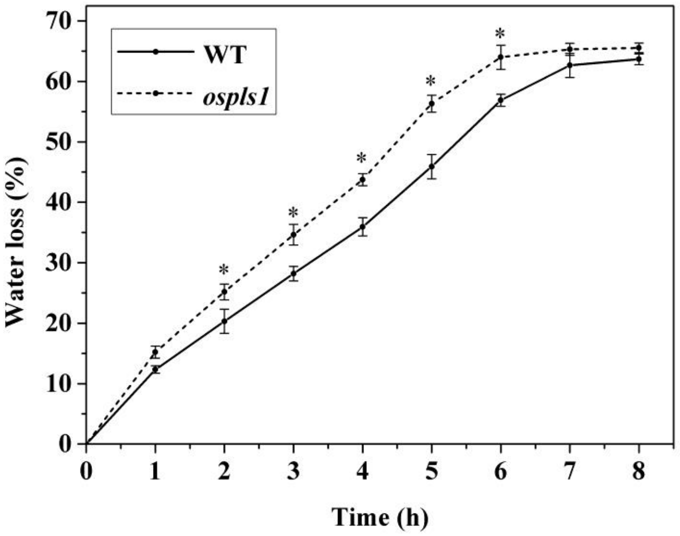

Fig. S4

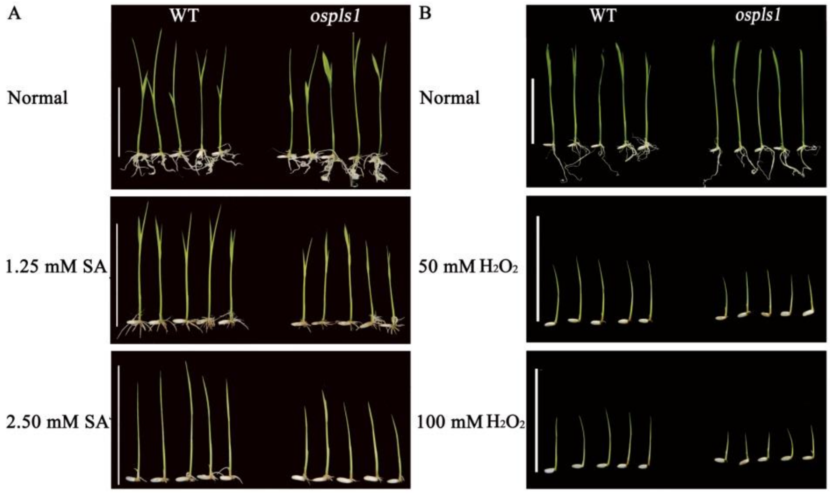
A
WT
ospls1
Normal
1.25 mM SA
2.50 mM SA
B
WT
ospls1
Normal
50 mM H2O2
100 mM H2O2

Fig. S5

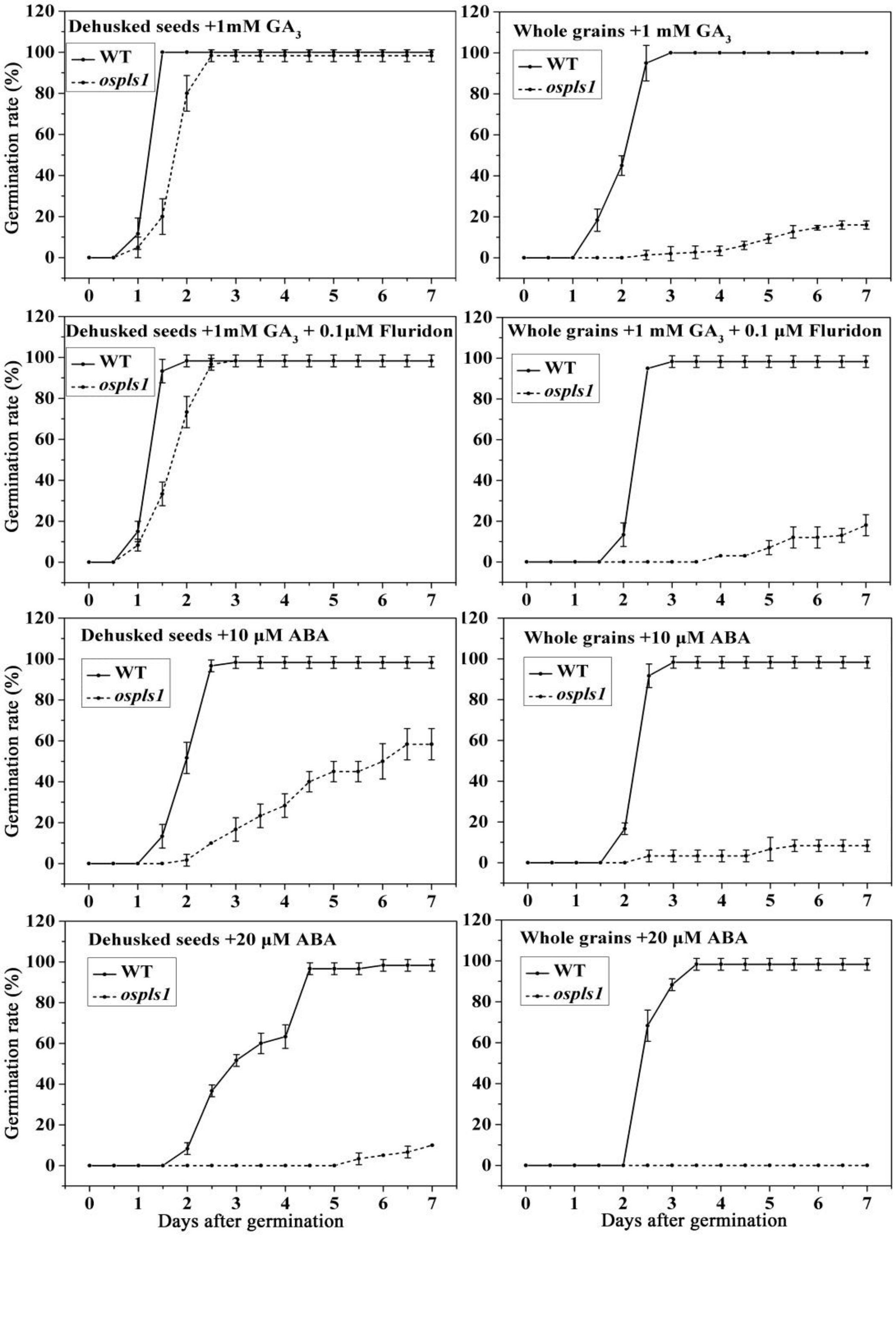
Dehusked seeds +1mM GA3
Whole grains +1 mM GA3
Dehusked seeds +1mM GA3 + 0.1μM Fluridon
Whole grains +1 mM GA3 + 0.1 μM Fluridon
Dehusked seeds +10 μM ABA
Whole grains +10 μM ABA
Dehusked seeds +20 μM ABA
Whole grains +20 μM ABA
WT
ospls1
Germination rate (%)
Days after germination
